# Hepatobiliary Cystadenomas and Cystadenocarcinoma. Report of Five Cases

**DOI:** 10.1155/1996/36975

**Published:** 1996

**Authors:** E. Gadijev, V. Ferlan-Marolt, J. Grkman

**Affiliations:** 1 Medical Centre Ljubljana Dept. of Gastroenterologic Surgery Slovenia; 2 Medical Faculty University of Ljubljana Institute of Pathology Slovenia

## Abstract

Clinicopathologic correlation offive cases ofcystadenomas of the liver are reported. All patients were female and radical surgical procedure, total excision or resection was performed four times, and partial excision once. In all patients the postoperative course was uneventful and they are all alive and well. Examined by conventional histological and special immuno-histochemical stains the tumors fulfilled diagnostic criteria for these rare cystic growths. The cyst wall was composed of three, histologically distinct layers. From the viewpoint of histogenesis and differential diagnosis immunohistochemical properties were analysed. CEA and EMA were demonstrated in epithelial cells and Vimentin in stromal cells.


*HPB Surgery*, 1996, Vol. 9, pp. 83–92
Reprints available directly from the publisher
Photocopying permitted by license only

© 1996 OPA (Overseas Publishers Association)
Amsterdam B.V. Published in The Netherlands
by Harwood Academic Publishers GmbH
Printed in Malaysia

### Hepatobiliary Cystadenomas and Cystadenocarcinoma. Report of Five Cases

E. GADŽIJEV, V. FERLAN-MAROLT* and J. GRKMAN

Medical Centre Ljubljana, Dept. of Gastroenterologic Surgery, Slovenia, *Medical Faculty, University of Ljubljana, Institute of Pathology, Slovenia

*(Received February 18, 1994)*

Clinicopathologic correlation of five cases of cystadenomas of the liver are reported. All patients were female and radical surgical procedure, total excision or resection was performed four times, and partial excision once. In all patients the postoperative course was uneventful and they are all alive and well. Examined by conventional histological and special immuno-histochemical stains the tumors fulfilled diagnostic criteria for these rare cystic growths. The cyst wall was composed of three, histologically distinct layers. From the viewpoint of histogenesis and differential diagnosis immunohistochemical properties were analysed. CEA and EMA were demonstrated in epithelial cells and Vimentin in stromal cells.

KEY WORDS: Liver cystadenoma cystadenocarcinoma surgery histology immunohistology.

### INTRODUCTION

During the last years surgical and radiological reports of Hepatobiliary Cystadenomas (HBC) have been increasing in the literature. Like other hepatic lesions, HBC are more frequently recognized because of improved imaging techniques, wide spread use of ultrasonography and also because of increased awareness of this entity[1].

HBCs are rare benign cystic tumors occurring in the liver parenchyma and bile ducts making up only about 5 per cent of hepatic cystic lesions[2]. They are found in the right and left lobe of the liver in approxi-mately equal numbers, in 15 per cents in both lobes and in 2 per cents in quadrate or caudate lobe[3]. HBCs are found most often in middle-aged women and have a strong tendency to recur after partial excision[4,5,6,7]. Macroscopically they often have a globular shape with a smooth external surface, under which there are multiple cystic cavities of various size containing mucinous fluid, divided by irregularly thick walls[2,8,6]. Microscopically HBCs have an inner layer of mucin secreting cuboidal or columnar epithelium with polypoid or papillary infoldings. The outer layer represents collagenous connective tissue[2,6]. In HBCs with mesenchymal stroma, a well defined clinico-pathologic entity occurring exclusively in young women, the locules are lined by three layers. Between the inner and outer lining an intermediate layer of undifferentiated mesenchymal cells exists[7,6]. In rare cases occuring only in males, acidophilic cell cystadenoma without mesenchymal stroma appears that is lined by eosinophilic cells resembling hepatocytes and could be classified as a semimalignant histological variant or a low-grade carcinoma[9].

Hepatobiliary cystadenomas are supposed to arise from foci of primitive hepatobiliary cells, or from normal intrahepatic ducts, sometimes from cholangiolar ducts in response to various stimuli[6]. The same stimulating factors may give rise to cystadenomas in pancreas[10] or kidneys[2].

Clinical presentations of HBCs are usually nonspecific[11] like abdominal discomfort, dull pain in the upper abdomen, nausea and anorexia, and sometimes a hepatic mass palpated[2,4,12,7,6]. Normal liver function tests accompany this tumor[4,6]. Jaundice or elevated alkaline phosphatase and bilirubin can be found in cases of external compression of the hepatic or extrahepatic biliary ducts[7,6].

*Address for correspondence:* Eldar Gadzijev, Medical Centre, Department of Gastroenterologic Surgery, Zaloska 7, 61000 Ljubljana, Slovenia.

Diagnosis of HBC can be reliably made on ultrasonography which shows anechoic ovoid or globular fluid-filled multilocular area with irregular margins, septal projections and mural nodules[8,5,13]. Exceptionally, intramural and septal calcifications may be present on ultrasonography and CT scans[5,12,14,15,13,16,11]. Computed tomography shows comparable abnormalities as ultrasonography but using contrast the wall and soft tissue elements in locular septa are enhanced[15,17]. Endoscopic retrograde cholangiography can show displacement of the bile ducts by the tumor but connections between the cyst and large intrahepatic bile ducts are usually not observed[4,18].

Complications like cholestasis due to compression of the bile duct confluence or common bile duct, intracystic haemorrhage, bacterial infection and rupture may be observed[19,6,4]. Recurrence after partial excision and transformation into cystadenocarcinoma are also mentioned as complications[4,7,2,1]. HBCs with mesenchymal stroma (CMS) have a special premalignant potential to transfer into carcinoma[7,4,6,18] while no clear evidence exists concerning the malignant evolution from cystadenoma without mesenchymal stroma[6].

Wheeler and Edmondson reported a case with additional cystadenoma in the left hepatic and common hepatic bile ducts causing obstructive jaundice[7]. Akwary with coworkers published a case with two simultaneous HBCs—one between the falciform ligament and the gallbladder, and another in the lumen of the common bile duct[6].

Hepatobiliary cystadenocarcinoma (HBCCa) usually develops in a pre-existing HBC[4,7]. Malignant epithelial transformation overlaying papillary proliferations of HBCs appears during a longer period of 6 years and more[20,7]. Kawarada proposed to classify it as cystadenocarcinoma with cystadenoma[21]. Cystadenocarcinoma may also arise de novo from the intrahepatic bile ducts forming multilocular cysts lined by mucus secreting epithelium with papillary infoldings[7,21]. Such cystadenocarcinomas are without mesenchymal stroma and have different sex incidence[2,7].

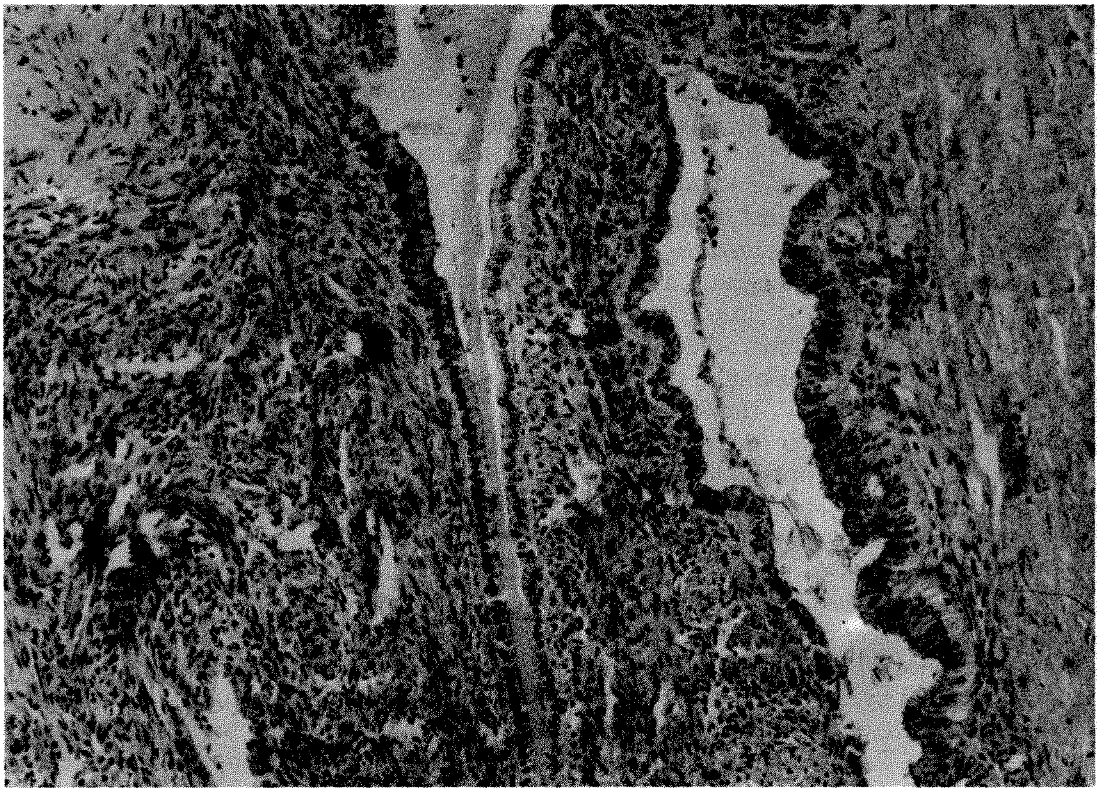

**Figure 1** Case 1: Benign area of HBCCa. Intercystic septa have columnar and cuboidal epithelium overlying a dense, highly cellular stroma. (H&E. Orig. magn. × 33).

### CASE REPORTS

Case 1: 58-year-old female with previously diagnosed asymptomatic gall stones was admitted to our hospital because of two episodes of right upper abdominal pain, irradiating to the back with nausea and vomiting. Abdominal ultrasound revealed thickened gall bladder wall and fatty liver. Gastroduodenoscopy discovered duodenal displacement. On admission, a palpable epigastric mass was found. Serum glucose and lactic dehydrogenase were elevated while other laboratory tests were normal. At operation, a big tumor spreading from the gallbladder to the stomac was found. At intraoperative ultrasound examination no metastases were found in the liver and no spread to the other abdominal organs was found at exploration. En bloc resection of the tumor together with bi-segmentectomy IV, V, resection of the stomac and lymphadenectomy was performed. Postoperative course was uneventfull. One year after the surgical procedure, the patient was well and without any signs of the disease.

*Pathomorphology:* The tumor was externally lobulated and consisted of greyyelowish solid areas which included cystic branching cavities. Necroses and hemorrhages alternated with gelatinous changes. Histologically, the cystic spaces had a distinct lining. Mucin producing columnar epithelial cells were arranged over a layer of dense vascular, somewhere highly cellular mesenchymal stroma. These structural elements formed intertumorous septa (Figure 1). Beside transition of benign epithelium to dysplasic and malignant cells, evident areas of malignant tubular formations creating papillary adenocarcinoma were observed. These structures showed positive, immunohistochemical staining reaction with EMA. CEA and wide-spectral keratin. The diagnosis was cystadenocarcinoma.

Case 2: A 30-year-old woman became our patient after an asymptomatic liver lesion had been incidentally detected with US investigation. She had not have any abdominal trauma and no history of parasytic infection. All laboratory tests were within normal ranges. On admission, ultrasound investigation was repeated and 100 ml of clear fluid was evacuated by puncture. At operation, a thin walled cyst occupying segments II and III was found. A deroofing was performed and the

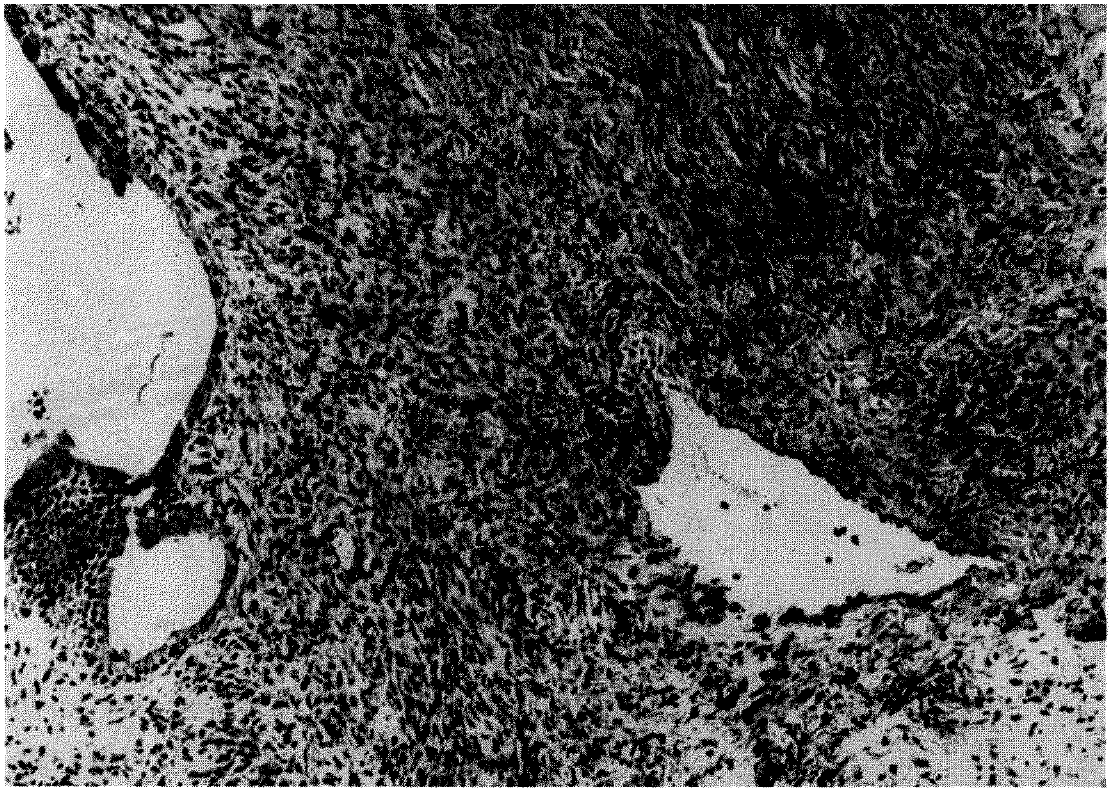

**Figure 2** Case 2: The intercystic septum with rather typical stroma has a flattened cuboidal epithelium in some areas. (H&E. Orig. magn. × 33).

rest of the cyst was treated with absolute alcohol. Postoperative period was uneventfull.

*Pathomorphology:* The external surface of the cyst was smooth and sharply demarkated from surrounding liver, the internal surface was rough with flattened tissue projections. Histologically, an undulating epithelial configuration was predominantly lined by cuboidal cells, but focally this layer was missing (Figure 2). Typical columnar epithelium with mucin covered some other areas. Dense stroma included spindled cells of mesenchymal origin, hyaline changes in some areas, and islets of oval and polygonal cells resembling hepatocytes. Mesenchymal stroma was vimentin positive.

Two years after operation she came back with upper abdominal discomfort and US revealed 5 × 7 cm cystic, partly solid lesion in the left subphrenic space. She was reoperated but no cystic formation was found. Omentum was adherent to the remnant tissue of cystadenoma. Total excision from liver was performed and a piece of omentum removed.

Characteristic histomorphological elements in the tumor remnant confirmed the diagnosis of cystadenoma. No signs of malignant transformation were observed.

Case 3: 25-year-old female presented with dull epigastric and right subcostal pain. A tender mass was palpated in the upper abdomen. In the enlarged left liver ultrasound revealed a huge cystic formation (17.5 × 14 × 13 cm) with thick wall enclosing thin septa. Angiography revealed lateral displacement of the left and right hepatic arteries because of this centrally located hypovascularized tumor. The portal vein was imperfectly presented probably due to compression. Alkaline phosphatase and transaminases were slightly elevated. CEA was 6 ng/ml and all other laboratory tests were normal. At operation, a large cyst was found growing in liver parenchyma from the segments I, IV, VI and VII, with a diameter of 17 cm. Intraoperative ultrasound revealed anterior displacement of vascular structures belonging to the right liver. 1500 ml of clear mucinous fluid was evacuated from the cyst and a part of the cyst wall was histo-logically examined. Frozen section investigation deter-mined the lesion as cystadenoma. Total cystectomy was performed using ultrasonic dissector (Figures 3 and 4). The residual cavity was filled with omentum. Postoperatively, a prolonged abdominal discharge occurred with a drain inserted in the cavity for 10 days. The patient was discharged on the 14th

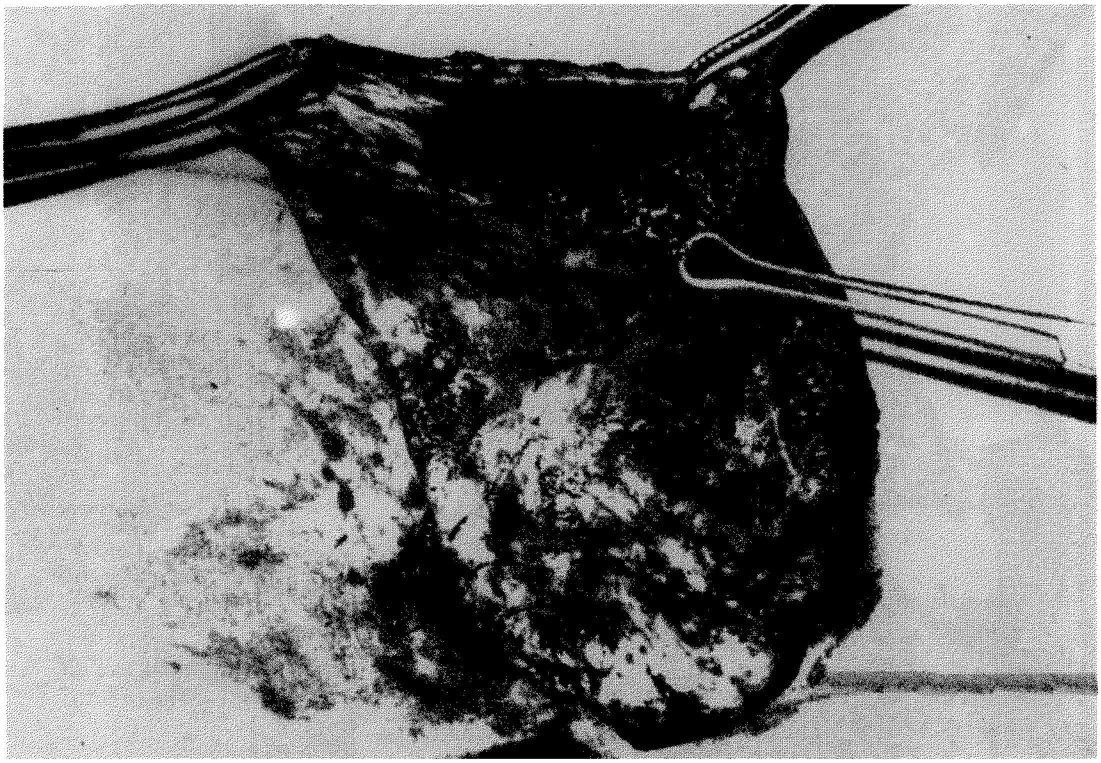

**Figure 3** Resected biliary cystadenoma in case 3.

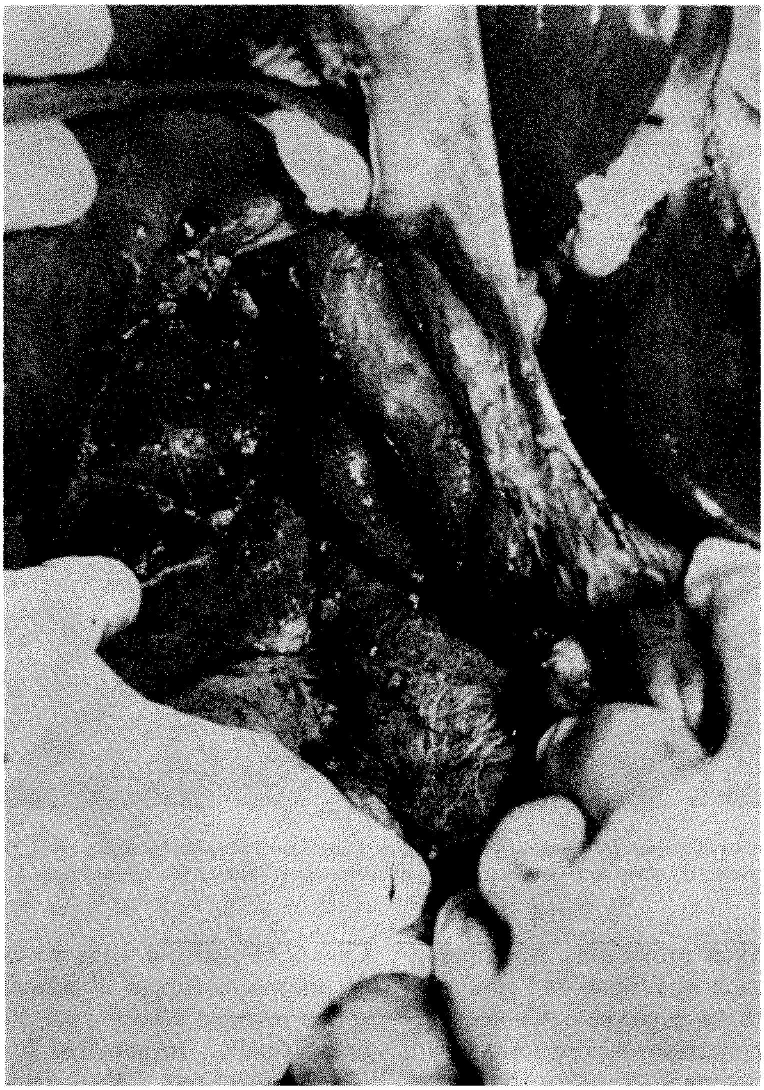

**Figure 4** Situation after excision of biliary cystadenoma from the liver in case 3.

postoperative day. 12 months later the patient felt healthy, and no signs of recurrence were clinically detected.

*Pathomorphology:* The multilocular tumorous lesion containing mucinous and gelatinous fluid compressed the adjacent liver. Histologic examination revealed variously dilated cavities with papillary septa (Figure 5). These tissue protrusions had tall columnar epithelial lining resting on the mesenchymal tissue layer. The latter contained cholesterol clefts and calcinations in some areas, and macrophages with siderin mixed with polymorphous inflammatory cells in the others.

Case 4: 57-year-old woman complaining of periodic, right upper abdominal pain was clinically examined. Beside gall stones, a cystic formation (10 × 7 cm), including numerous small cysts were discovered in the left liver lobe. Although history and serology for hydatid disease was negative the space occupying lesion was assumed to be a parasitic cyst. On admission, a large mass was palpated in the epigastrium, gamma glutamyl transpeptidase was slightly elevated, but other laboratory tests were normal. At operation, a cyst with a firm greyish wall was found in segment IVa. When opening the cyst 100 ml of mucinous fluid was evacuated. A part of the cyst contained papillary

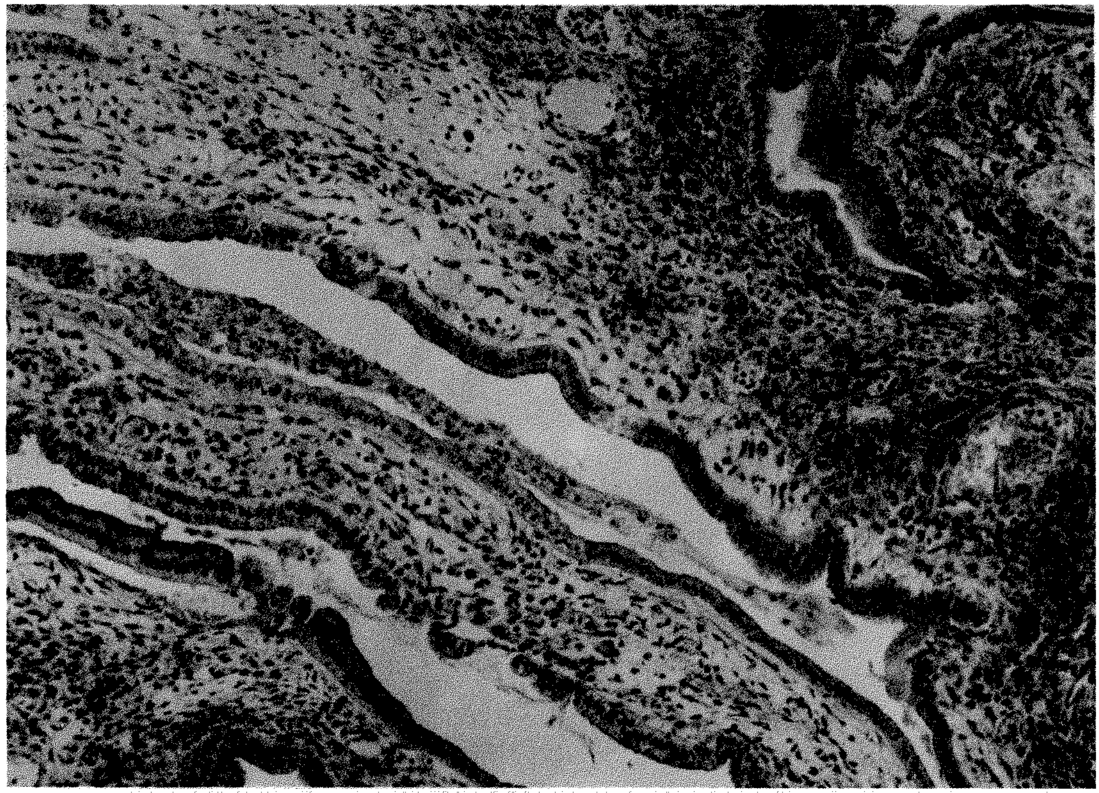

**Figure 5** Case 3: Cystic cavities of various forms and sizes are lined by a single layer of epithelial cells with uniform nuclei and rare mitoses. Dense cellular, as well as moderately collagenous stroma includes inflammatory cells and a few siderophages. (H&E. Orig. magn. × 33)

infoldings and small tissue projections. A communication with biliary system was found and confirmed with intraoperative cholangiography. Cholecystectomy, as well as total cystectomy was performed with ligation of the bile duct communicating with the cyst. Cystectomy was performed using ultrasonic disector so that liver structures on the both liver sides were preserved intact.

The postoperative course was uneventfull and the patient was discharged from the hospital eight days after operation. 10 months after operation she was well.

*Pathomorphology*: The cystic wall was rather thick, interwoven with fibrous septa. Histologically, the surface of the wall was covered with mucin producing columnar epithelium spreading over collagenous tissue (Figure 6). This firm tissue included haemorrhages and necroses, surrounded with chronic inflammatory cells and macrophages with sider in or biliary pigment. No parasites could be identified in the tissue material, and no characteristic histology induced by parasites could be detected. The cyst was ranged to biliary cystadenoma.

Case 5: 61-year-old woman was investigated because of nonspecific upper abdominal discomfort. Ultrasound revealed a large cyst, 10 cm in diameter with many smaller, presumably hydatid daughter cysts inside the big one. CT confirmed the former investigation and located the cyst between the right and left liver lobes. Since the diagnosis seemed certain, no biopsy was performed. On admission, the blood tests showed raised values of bilirubin, alkaline phosphatase (fivefold), gamma glutamyl transpeptidase (tenfold), as well as transaminases (threefold), ammonia, blood sugar, cholesterol, and accelerated sedimentation rate.

At operation, small fibrotic liver with cystic formation in segment IVb was found. Gall bladder was normal while common hepatic duct was dilated, measuring 2.5 cm. Intraoperative ultrasound revealed a cyst with internal echoes not quite typical for hydatid daughter cysts. During cholecystectomy the common hepatic duct was opened. An ovoid alteration in its lumen resembling a daughter hydatid cyst was fixed to the wall and protruding into the left hepatic duct. Surgical extraction was impossible, therefore. A tissue

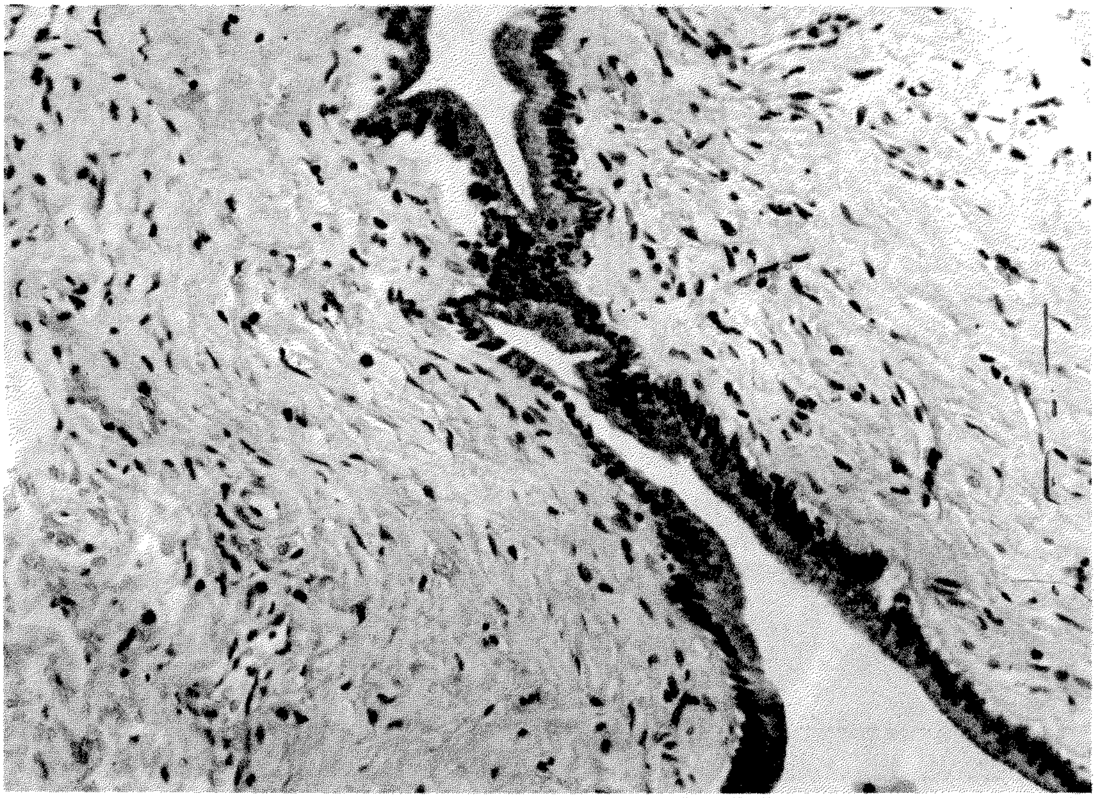

**Figure 6** Case 4: Tall columnar epithelium covers collagenous areas in the wall of the cyst. (H&E. Orig. magn. × 66).

specimen was cut for frozen section investigation which revealed a cystadenoma. Left hemihepatectomy with hepatocholedochus resection was accomplished and a cholangiojejuno anastomosis to the anterior and posterior sector ducts performed. Postoperative course was uneventfull. The patient was discharged eight days after operation. Thirteen months after operation she was well and without any recurrence.

*Pathomorphology*: The main characteristic of the lesion were polypoid and papillary projections in the multilocular cavity. The typical epithelial cells rested on a thin basement membrane (Figure 7). The sub-epithelial stroma included focal hyaline changes, and mild inflammatory infiltrations. Necroses and haemorrhages were noted, too. The cyst was identified as biliary cystadenoma.

### DISCUSSION

The majority of published cases of HBC are females[17] as in our report, where all the patients were female 25 to 61 years old. Males outnumbered females only in Japanese series[21]. Three of our cases were presented with upper abdominal pain or abdominal discomfort, the symptoms found also in other reported cases[1,2,6,7]. Abdominal distress was much more expressed in the patient with cystadenocarcinoma whose pain was irradiating into back with concomitant nausea and vomiting. In one case, the cystic tumor was found just incidentally during US investigation. Three times the tumor was palpable in upper abdomen: in the case of cystadenocarcinoma of the gall bladder (case 1) and twice in the patients where the cystic lesion was rather large (cases 3 and 5). US investigation appeared to be a reliable method to discover these tumors in our series. US revealed a cystic lesion in the liver in four of our cases, and an altered, thick-walled gallbladder in the case of cystadenocarcinoma.

Macroscopical differential diagnosis between HBC and simple cyst of the liver is not difficult with the latter not having septations, papillary projections and mucinous fluid[4]. Sometimes it is more difficult to distinguish HBC as well as HBCCa from hydatid cyst, abscess, haematoma, cystic hamartoma or necrotic neoplasm[8].

Because of nonspecific clinical signs and symptoms, the preoperative diagnosis depends on imaging find-

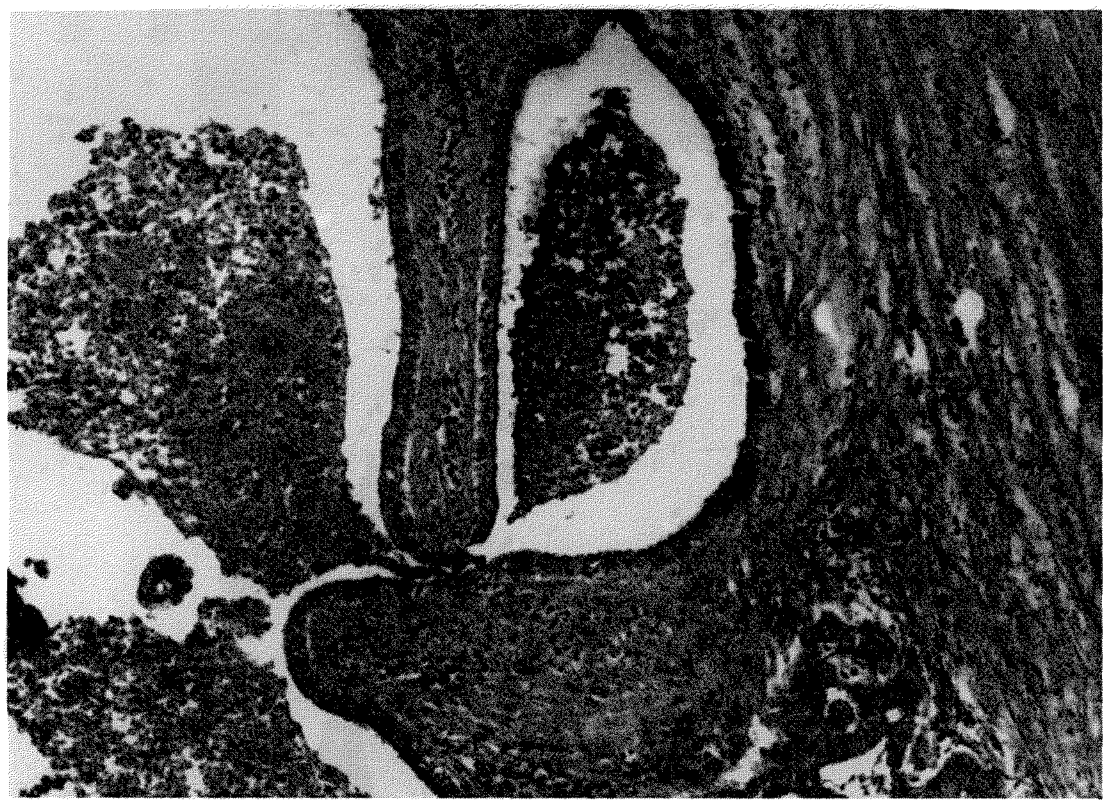

**Figure 7** Case 5: Polypoid projections in the multilocular cavity are characterized with columnar epithelial lining and include hyaline stromal layer with sparse inflammatory infiltration. (H&E. Orig. magn. × 33).

ings. CT and US are both the most useful imaging methods to detect these tumors[15,17], as well as to establish their macroscopic features the correct diagnosis depends on.

Our US investigations declared the cysts as parasitic in three cases (cases 3, 4, 5), and for a simple biliary cyst in one (case 2). According to the US findings a cystadenoma could have been suspected in three cases. Sonographic diagnostic problems appear when solitary hydatid cysts with detached hydatid membrane, daughter cysts, and hydatid debris with calcifications are observed. They might give similar sonographic picture to architectural elements of HBC and HBCCa[4,5]. To localize the lesion more precisely before the operation, additional CT was used in one case of ours (case 4). Because of the central position of the cyst in the liver we used angiography in one case (case 3).

Liver function tests were pathological in the patient with cystadenoma protruding into the hepatic duct (case 5), in the patient with communication of the HBC with biliary tree (case 4) and in the patient with the largest centrally located, HBC. The uncommon communication of HBC with the biliary tree, described in an article by Pinson[1], was found in our case 4. We confirmed it with intraoperative cholangiography, but could not prove whether biliary communication was a primary lesion, or the consequence of bile duct wall compression with the cyst. In our case 5 a tumorous protrusion into the left and common hepatic ducts was found. The finding of the cyst in biliary ducts might indicate that it was of biliary origin or it represented a cystic duplication of the ducts. In the available literature no similar case was reported. An additional HBC was found and described in biliary tree as a separate coexisting tumor[6,7].

Our case of HBCCa with mesenchymal stroma which arose in the gall bladder is a particular example of our series. A similar case with operation has not been reported yet. The broad term of cystadenocarcinoma of the liver requires a more detailed classification based on histological determination of these malignant cystic tumors as suggested by Kawarada and coworkers[21]. According to the presumptive origin, cystadenocarcinoma and carcinoma arising from a simple liver cyst should be classified as separate pathological entities[22]. Immunohistochemical confirmation of these tumors is necessary since between otherwise benign histological elements some premalignant or already carcinomatous changes could exist[23,24]. Our case of HBCCa had a macroscopic appearance of a malignant tumor different from usual gallbladder carcinomas. It was invasively growing in the stomach. Histologic findings confirmed HBCCa.

HBCs, often misinterpreted as simple cysts, have been treated by aspiration and sclerosis, marsupialisation, internal Roux-en-Y drainage or partial excision which have been associated with a lot of complications as well as recurrences[1,4]. To prevent recurrences and/or malignant alteration HBCs have to be treated with total excision or sometimes by hepatic resection[1,2,4,6,7,12,21,25]. Surgical intervention, especially total excision is ideal because it offers the best chances of cure and might be the only definite therapy[26,27]. Besides it permits a proper histological examination of the cyst, and a detailed classification. However, the general condition of the individual patient, anatomic position of the cysts, and the experience of the surgeon should determine the final therapeutic decision[28]. Partial excision should only be reserved for those cases in which total excision might jeopardize the main liver structures[6]. When HBCCa is suspected and proved by histological examination during the operation, a liver resection should be performed, and better results as in cases of HCC could be reliably expected[11,19,20].

In our cases of HBCs total excision was performed three times. In two cases (cases 3, 5), pathomorphological analysis of the frozen sections revealed HBC, and in case 4 where the biliary communication with the cyst was the reason for total excision. In one case supposed to be a simple cyst excision of the cyst roof and sclerosation was the only procedure performed. Later pathohistological examination discovered HBC in this tissue material. The patient returned with a lesion found on US investigation thought to be a recurrent tumor two years later. The patient with HBCCa proven with pathomorphological examination was treated with radical resection extended to the stomach and lymphadenectomy.

There was no operative or hospital mortality among our patients. No complications were observed except in case where abdominal drainage had to be prolonged for ten days.

We believe that surgeons must think about HBC and HBCCa, when they find cystic lesions in the liver. Puncture with sclerosation should be used as a therapeutic intervention only if the diagnosis of HBC is excluded. As for the rest, total excision of the tumor is the most successful therapeutic method and the procedure of choice.

### REFERENCES

1. Pinson, C. W., Munson, J. L., Rossi, R. L. and Braasch, J. W. (1989) Enucleation of intrahepatic biliary cystadenoma. *Surgery Gynecology Obstetrics*, **169**, 535–537.
2. Ishak, K. G., Willis, G. W., Cummins, S. D. and Bullock, A. A. (1977) Biliary cystadenoma and cystadenocarcinoma: Report of 14 cases and review of the literature. *Cancer*, **38**, 322–338.
3. Scully, R. E., Mark, E. J. and McNeely, B. U. (1985) Weekly clinicopathological exercises. *New England Journal of Medicine*, **313**, 1275–1281.
4. Benhamou, J. P. and Menu, Y. (1988) Non parasitic cystic diseases of the liver and intrahepatic biliary tree. *In Surgery of the Liver and Biliary Tract*, edited by L. H. Blumgart, vol. 2. pp 1013–1023 Edinbourgh—London, Melbourne and New York: Churchill Liwingstone.
5. Korobkin, M., Stephens, D. H., Lee, J. K. T., Stanley, R. J., Fishman, E. K., Francis, I. R., Alpern, M. B. and Rynties, M. (1989) Biliary Cystadenoma and Cystadenocarcinoma: CT and Sonographic Findings American Journal of Radiolory, **153**, 507–511.
6. Akwari, O. E., Tucker, A., Seigler, H. F. and Itani, K. M. F. (1990) Hepatobiliary Cystadenoma with Mesenchymal Stroma. *Annals of Surgery*, **211**, 18–27.
7. Wheeler, D. A. and Edmondson, H. A. (1985) Cystadenoma with Mesenchymal Stroma (CMS) in the Liver and Bile Ducts. *Cancer*, **56**, 1434–1445.
8. Forrest, M. E., Cho, K. J., Shields, J. J., Wicks J. D., Silver, T. M. and McCormick, T. L. (1980) Biliary cystadenomas: Sonographic-angiographic-pathologic correlations. *American Journal of Radiology*, **135**, 723–727.
9. Peters, R. L. (1986) Neoplastic diseases. In Liver Pathology edited by R. L. Peters and J. R. Craig, 1st edn, pp. 345–348, New York: Churchill Livingstone.
10. Keech, M. K. (1951) Cystadenomata of the pancreas and intrahepatic bile ducts. *Gastroenterology*, **19**, 568–574.
11. Marsh, J. L., Dahms, B. and Longmire Jr, W. P. (1974) Cystadenoma and cystadenocarcinoma of the biliary system. *Archives of Surgery*, **109**, 41–43.
12. Marcial, M. A., Hauser, S. C., Cibas, E. S. and Braver, J. (1986) Intrahepatic biliary cystadenoma. Clinical, radiological, and pathological findings. *Digestive Diseases in Science*, **31**, 884–888.
13. Carroll, B. A. (1978) Billary cystadenoma and cystadenocarcinoma: Gray scale ultrasound appearance. *Journal of Clinical Urology*, **6**, 337–340.
14. Stanley, J., Vujic, J., Schabel, S. I., Gobein, R. P. and Reines, H. D. (1983) Evaluation of biliary cystadenoma and cystadenocarcinoma. *Gastrointestinal Radiology*, **8**, 245–248.
15. Choi, B. I., Lim, J. H., Han, M. C., Lee, D. H., Kim, S. H., Kim, Y. I. and Kim, C. W. (1989) Biliary cystadenoma and cystadenocarcinoma: CT and sonographic findings. *Radiology*, **171**, 57–61.
16. Short, W. F., Nedwich, A., Levy, H. A. and Howard, J. M. (1971) Biliary cystadenoma: report of a case and review of the literature. *Archives of Surgery*, **102**, 78–80.
17. Cheung, Y. K., Chan, F. L., Leong, L. L. Y., Collins, R. J. and Cheung, A. (1991) Biliary Cystadenoma and Cystadenocarcinoma: Some Unusual Features. *Clinical Radiology*, **43**, 183–185.
18. Williams, J. G., Newman, B. M., Sutphen, J. L., Madison, J., Frierson, H. and McIlhenny, J. (1990) Hepatobiliary cystadenoma: A rare hepatic tumor in child. *Journal of Pediatric Surgery*, **25**, 1250–1252.
19. Roekel van V., Marx, W. J. and Greenlaw, R. L. (1982) Cystadenoma of the liver. *Journal of Clinical Gastroenterology*, **4**, 167–172.
20. Woods, G. (1981) Biliary cystadenocarcinoma. Case report of hepatic malignancy originating in benign cystadenoma. *Cancer*, **47**, 2936–2940.
21. Kawarada Y., Taoka, H. and Mizumoto, R. (1991) A report of 5 cystic bile duct carcinoma of the liver and proposal of a new classification. *Gastroenterologia Japonica*, **26**, 80–89.
22. Devine, P. and Ucci, A. A. (1985) Biliary cystadenocarcinoma arising in a congenital cyst. *Human Pathology*, **16**, 92–94.
23. Thomas, J. A., Scriven, M. W., Puntis, M. C. A., Jasani, B. and Wiliams, G. T. (1992) Elevated serum CA 19–9 levels in hepatobiliary cystadenoma with mesenchymal stroma. Two case reports with immunohistochemical confirmation. *Cancer*, **70**, 1841–1846.
24. Gourley, W. K., Kumar, D., Bouton, M. S., Fish, J. C. and Nealon, W. (1992) Cystadenoma and cystadenocarcinoma with mesenchymal stroma of the liver. Immunohistochemical analysis. *Archives of Pathology and Laboratory Medicine*, **116**, 1047–1050.
25. Forde, K. A., Wolf, M., Fuld, S. D. and Price, J. B. (1974) Hepatic lobectomy for biliary cystadenoma. *American Journal of Surgery*, **40**, 647–650.
26. Cahill, C. J., Bailey, M. E. and Smith, M. G. H. (1982) Mucinous cystadenomas of the liver. *Clinical Oncology*, **8**, 171–177.
27. Edwards, J. D., Eckhauser, F. E., Knoll, J. A., Strodel, W. E. and Appelman, H. D. (1987) Optimizing surgical management of symptomatic solitary hepatic cysts. *American Journal of Surgery*, **53**, 510–514.
28. Henne-Bruns, D., Klomp, H. J. and Kremer, B. (1993) Nonparasitic liver cysts and polycystic liver disease: results of surgical treatment. *Hepatogastroenterology*, **40**, 1–5.

